# Creatinine-to-body weight ratio is a predictor of incident diabetes: a population-based retrospective cohort study

**DOI:** 10.1186/s13098-021-00776-8

**Published:** 2022-01-15

**Authors:** Jiacheng He

**Affiliations:** Emergency Department, Nanhai District People’s Hospital Of Foshan, Foshan, 528200 China

**Keywords:** Type 2 diabetes, Creatinine, Body weight, Muscle mass, Insulin resistance, Skeletal muscle

## Abstract

**Purpose:**

Creatinine to body weight (Cre/BW) ratio is considered the independent risk factor for incident type 2 diabetes mellitus (T2DM), but research on this relationship is limited. The relationship between the Cre/BW ratio and T2DM among Chinse individuals is still ambiguous. This study aimed to evaluate the correlation between the Cre/BW ratio and the risk of T2DM in the Chinese population.

**Methods:**

This is a retrospective cohort study from a prospectively collected database. We included a total of 200,658 adults free of T2DM at baseline. The risk of incident T2DM according to Cre/BW ratio was estimated using multivariable Cox proportional hazards models, and a two-piece wise linear regression model was developed to find out the threshold effect.

**Results:**

With a median follow-up of 3.13 ± 0.94 years, a total of 4001 (1.99%) participants developed T2DM. Overall, there was an L-shaped relation of Cre/BW ratio with the risk of incident T2DM (P for non-linearity < 0.001). When the Cre/BW ratio (× 100) was less than 0.86, the risk of T2DM decreased significantly as the Cre/BW ratio increased [0.01 (0.00, 0.10), P < 0.001]. When the Cre/BW ratio (× 100) was between 0.86 and 1.36, the reduction in the risk of developing T2DM was not as significant as before [0.22 (0.12, 0.38), P < 0.001]. In contrast, when the Cre/BW ratio (× 100) was greater than 1.36, the reduction in T2DM incidence became significantly flatter than before [0.73 (0.29,1.8), P = 0.49].

**Conclusion:**

There was an L-shaped relation of Cre/BW ratio with incidence of T2DM in general Chinese adults. A negative curvilinear association between Cre/BW ratio and incident T2DM was present, with a saturation effect predicted at 0.86 and 1.36 of Cre/BW ratio (× 100).

**Supplementary Information:**

The online version contains supplementary material available at 10.1186/s13098-021-00776-8.

## Background

Diabetes, an ever-growing health problem, places a heavy economic burden on individuals and society [1–4]. China has the heaviest burden of diabetes mellitus worldwide with nearly a quarter of the world's diabetes patients living in China. In 2013, the prevalence of adult diabetes mellitus was 10.4% [5, 6]. Therefore, prevention and treatment of type 2 diabetes (T2DM) is important.

Previous studies have shown that obesity is a significant risk factor for diabetes [7–9]. Diabetes accelerates muscle mass through hyperglycemia, insulin resistance (IR), and inflammatory cytokines [10]. Furthermore, a recent study showed that decreased muscle mass is closely related to IR [11]. Therefore, muscle mass is an important target for the prevention and treatment of T2DM.

Studies have revealed that weight mass-adjusted skeletal muscle mass index (SMI), defined as skeletal muscle mass/weight, is a risk factor for T2DM [12]. Creatinine (Cre) is the only metabolite of phosphate creatine in the body’s skeletal muscles. Cre, although a marker of renal function, is affected by muscle size since the muscle mass produces Cre. Because the total skeletal muscle mass is generally stable, the Cre concentration is relatively stable [12]. Thus, Cre is also an inexpensive, quickly accessible alternative marker of muscle quality in individuals with normal renal functions [13]. Furthermore, in a recent study, the Cre-to-body weight (Cre/BW) ratio is closely related to the incidence of diabetes in Japanese participants [14]. However, the relationship between the Cre/BW ratio and T2DM has not been studied in Chinese participants. Our research goal was to evaluate the correlation between the Cre/BW ratio and the risk of developing incident T2DM using freely-downloaded data, in a secondary data analysis [15].

## Methods

### Data source

Data were freely downloaded from the DATADRYAD website (www.datadryad.org). In line with the Dryad Terms of Service, we obtained the Chen et al. [15] datasets on the association of body mass index (BMI) and age with incident diabetes in Chinese adults, from a prospectively collected database. The following variables were included in the dataset: sex, age, BMI, drinking status, smoking status, family history of diabetes, low-density lipoprotein cholesterol (LDL-C), high-density lipoprotein cholesterol (HDL-C), total cholesterol (TC), triglyceride (TG), fasting plasma glucose (FPG), Cre, aspartate aminotransferase (AST), alanine aminotransferase (ALT), systolic blood pressure (SBP), diastolic blood pressure (DBP), incident diabetes at follow-up, and follow-up time. In the original paper [15], the authors declared that they had relinquished copyright and relevant ownership of the database. Thus, this database can be used for secondary analyses without violating the rights of the authors.

### Study population

**Fig. 1:**
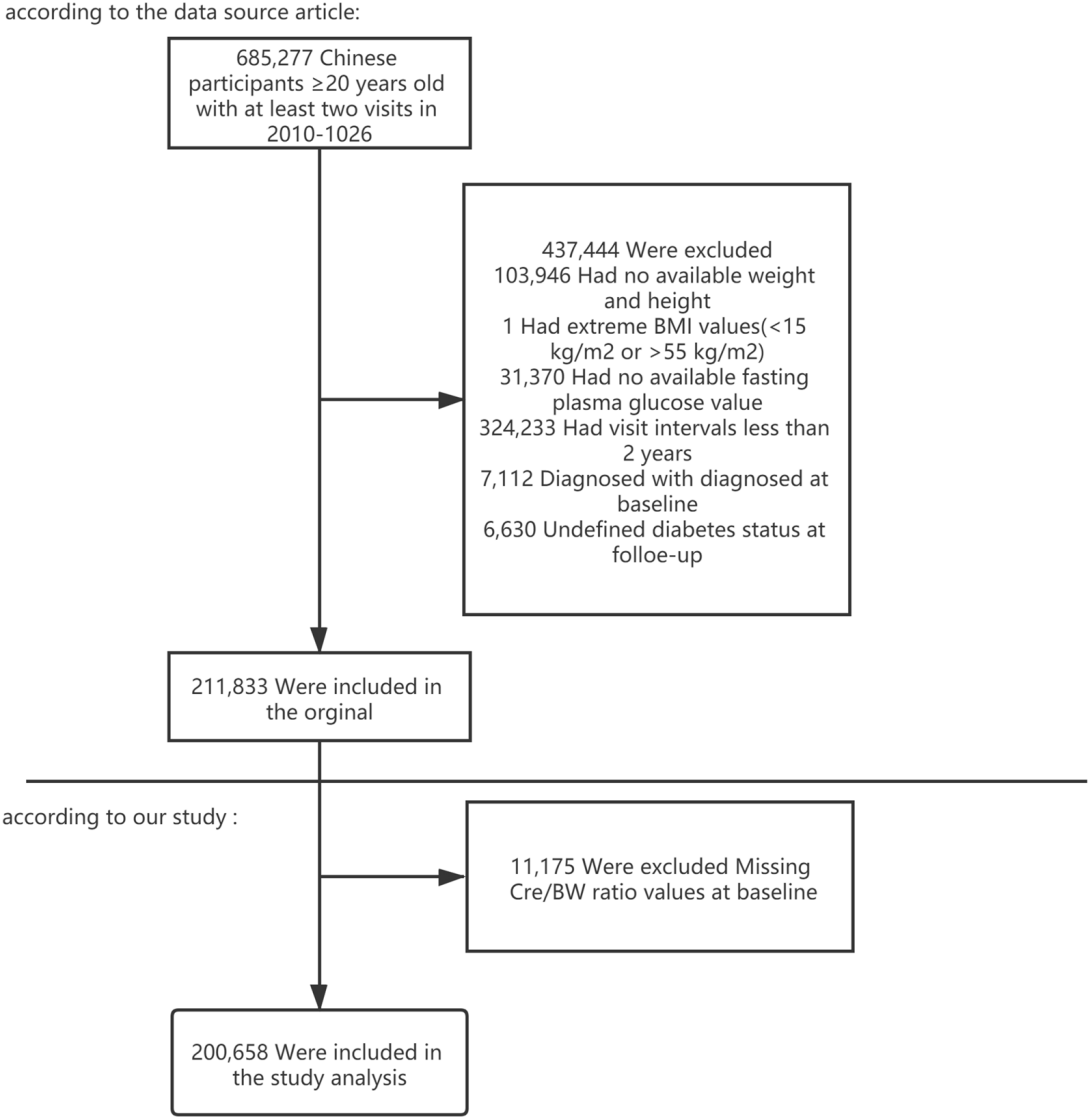
Flowchart of study participants

Data were obtained from a database provided by the Rich Healthcare Group in China. The present study enrolled 685,277 participants who received a health check-up, were at least 20 years old, and had records of at least two visits between 2010 and 2016 across 32 sites and 11 cities (Shanghai, Beijing, Nanjing, Suzhou, Shenzhen, Changzhou, Chengdu, Guangzhou, Hefei, Wuhan, Nantong) in China. The data we obtained were initially screened based on the following exclusion criteria: (1) no available information on weight, height, sex, and FPG at baseline; (2) extreme BMI values (< 15 or > 55 kg/m^2^); (3) participants with visit intervals less than two years; and (4) participants diagnosed with diabetes at baseline, and participants with undefined diabetes status at follow-up [15]. Finally, 211,833 participants were included in the analysis. The institutional ethics committee did not require any study approval or informed consent for the retrospective component of the research. Data of some participants were excluded from the cohort for further analysis as follows: those with missing Cre/BW ratios at baseline (n = 11,175). In total, 200,658 participants (110,431 men and 90,227 women) were included in the analysis (Fig. 1).

### Measurement of the Cre/BW ratio and other covariates

The researchers retrieved the data included in our retrospective cohort study. The study design for the primary study was documented elsewhere [15]. A detailed questionnaire was used to obtain demographic characteristics, lifestyle, disease history, and medical history. Height measurement was accurate to 0.1 cm. Weight measurement was accurate to 0.1 kg, and the participants were required to wear lightweight clothes and no shoes. The BMI was calculated as weight/height squared (kg/m^2^). Cre/BW ratio was calculated as Cre (mg/dL) divided by the weight (kg) [16]. Fasting venous blood was drawn to estimate serum LDL-C, TG, TC, HDL-C, FPG, blood urea nitrogen (BUN), Cre, ALT, and AST levels using an automatic biochemical analyzer (Beckman 5800). Because this was a retrospective cohort study, observation bias was naturally reduced.

### Ascertainment of diabetes diagnosis

Diabetes diagnosis was defined according to FPG ≥ 7.00 mmol/L or self-reported diabetes diagnosis. Ascertainment of diabetes was dependent on the participants’ date of diagnosis or the last visit.

### Statistical analysis

The missing values of the other variables were first imputed before the statistical analysis. The missing values for continuous variables (such as height, FPG, TC, TG, HDL-C, LDL, ALT, AST, BUN, SBP, and DBP, were imputed using the mean or median. Missing data on categorical variables (such as smoking and drinking status) were treated as a set of categorical variables [17]. Normally distributed continuous variables are presented as means with standard deviations (SDs), while those with skewed distribution are expressed as medians with interquartile ranges (IQRs). Categorical variables are presented as frequencies and percentages. We used the chi-square test, one-way analysis of variance, or Kruskal–Wallis test to examine the statistical differences between groups stratified by Cre/BW ratio quartiles. We employed univariate and multivariate Cox proportional hazard models to assess the relationship between the Cre/BW ratio and the risk of T2DM. Three models were constructed: model 1 (univariate), the crude model; model 2, adjusted for age and sex; and model 3, adjusted for age, sex, height, FPG, TC, TG, LDL, HDL-C, BUN, AST, ALT, SBP, DBP, drinking status, smoking status, and family history of diabetes. In the models, we used the median value of each quartile of the Cre/BW ratio to perform the linear trend tests: quartile 1 (Q1), quartile 2 (Q2), quartile 3 (Q3), and quartile 4 (Q4). In addition, restricted cubic spline Cox regression analysis with 7 knots (2.5th, 18.33rd, 34.17th, 50.00th, 65.83rd, 81.67th, and 97.50th percentiles of Cre/BW ratio) was performed to test for linearity and explore the shape of the dose–response relationship of Cre/BW ratio and incident T2DM. The threshold level of Cre/BW ratio was determined using a recurrence method, including the selection of the turning point along a predefined interval and the selection of the turning point that yielded the maximum likelihood model. Meanwhile, to better reflect the changes in the curve and the dose–response effect, we set two additional turning points for the line inflection analysis. A log-likelihood ratio test was used to compare the three-piecewise linear regression model with the one-line linear model separately. To identify modifications and interactions, we used a stratified Cox regression model and likelihood ratio test for the different subgroups according to sex, age (< 40, 40–60, and ≥ 60 years), smoking status (never, ever, current, or not recorded), drinking status (never, ever, current, or not recorded), family history of diabetes, and BUN (≤ 7.1 or > 7.1). We used R statistical software (R Foundation, Vienna, Austria) to analyze the data. Statistical differences were considered significant when the calculated P-value was less than 0.05.

## Results

### Baseline characteristics of the patients

Baseline characteristics of the selected participants according to the quartiles of Cre/BW ratio are presented in Table 1. A total of 200,658 participants (55.03% men and 44.97% women; mean age, 42.24 years) were included in this study. After an average follow-up of 3.13 ± 0.94 years, 4001(1.99%) participants developed T2DM Participants in the highest group of Cre/BW ratio (Q4) had lower weight, BMI, SBP, DBP, TC, TG, FPG, LDL, ALT, AST, and consisted of more males and drinkers than for the other groups (Q1-3). The Q4 participants had higher Cre, Cre/BW ratio, HDL-C, BUN, and more smokers and a family history of diabetes than those with in the Q1-Q3 groups.

**Table 1:** Baseline characteristics of study participants according to quartiles of Cre/BW ratio

	Overall	Q1(< 1.062)	Q2 (≥ 1.062 to < 1.219)	Q3 (≥ 1.219 to < 1.396)	Q4 (≥ 1.396)	P-value
Number	200,658	50,165	50,164	50,163	50,166	
Age (years)	42.24 ± 12.73	42.37 ± 11.69	42.11 ± 12.04	41.98 ± 12.67	42.50 ± 14.35	< 0.001
Height (cm)	166.50 ± 8.33	166.61 ± 8.68	166.63 ± 8.46	166.62 ± 8.23	166.13 ± 7.91	< 0.001
Weight (kg)	64.75 ± 12.23	70.98 ± 13.54	65.75 ± 11.73	63.08 ± 10.72	59.18 ± 9.40	< 0.001
BMI (kg/m^2^)	23.24 ± 3.34	25.43 ± 3.50	23.55 ± 2.95	22.62 ± 2.80	21.38 ± 2.66	< 0.001
SBP (mmHg)	119.04 ± 16.40	121.38 ± 16.81	118.77 ± 16.25	118.03 ± 15.90	117.98 ± 16.41	< 0.001
DBP (mmHg)	74.16 ± 10.81	75.65 ± 11.31	74.20 ± 10.83	73.68 ± 10.54	73.11 ± 10.36	< 0.001
FPG (mmol/L)	4.91 ± 0.61	4.99 ± 0.62	4.91 ± 0.61	4.88 ± 0.61	4.87 ± 0.61	< 0.001
TC (mmol/L)	4.72 ± 0.89	4.79 ± 0.91	4.73 ± 0.89	4.70 ± 0.89	4.64 ± 0.88	< 0.001
TG (mmol/L)	1.34 ± 1.03	1.51 ± 1.22	1.37 ± 1.04	1.30 ± 0.95	1.20 ± 0.83	< 0.001
Cre/BW ratio (× 100)	1.24 ± 0.27	0.94 ± 0.10	1.14 ± 0.04	1.30 ± 0.05	1.59 ± 0.22	< 0.001
HDL-C (mmol/L)	1.37 ± 0.23	1.36 ± 0.23	1.37 ± 0.23	1.38 ± 0.24	1.38 ± 0.23	< 0.001
LDL (mmol/L)	2.77 ± 0.52	2.79 ± 0.53	2.77 ± 0.52	2.77 ± 0.52	2.75 ± 0.51	< 0.001
ALT (U/L)	23.99 ± 21.98	27.78 ± 25.98	24.53 ± 22.77	22.96 ± 21.36	20.68 ± 16.03	< 0.001
AST (U/L)	24.11 ± 7.95	24.53 ± 7.84	24.12 ± 8.34	23.96 ± 7.43	23.82 ± 8.15	< 0.001
BUN (mmol/L)	4.66 ± 1.15	4.44 ± 1.10	4.58 ± 1.11	4.70 ± 1.12	4.92 ± 1.22	< 0.001
Cre (mg/dl)	0.79 ± 0.18	0.66 ± 0.13	0.75 ± 0.14	0.82 ± 0.14	0.94 ± 0.18	< 0.001
Develop T2DM						< 0.001
No	196,657 (98.01)	48,542 (96.76)	49,175 (98.03)	49,388 (98.46)	49,552 (98.78)	
Yes	4001 (1.99)	1623 (3.24)	989 (1.97)	775 (1.54)	614 (1.22)	
Sex (%)						< 0.001
Men	110,431 (55.03)	20,467 (40.8)	25,616 (51.06)	29,599 (59.01)	34,749 (69.27)	
Women	90,227 (44.97)	29,698 (59.2)	24,548 (48.94)	20,564 (40.99)	15,417 (30.73)	
Smoking status (%)						< 0.001
Current	11,373 (5.67)	2421 (4.83)	2817 (5.62)	2964 (5.91)	3171 (6.32)	
Ever	2473 (1.23)	505 (1.01)	596 (1.19)	677 (1.35)	695 (1.39)	
Never	43,816 (21.84)	10,504 (20.94)	10,942 (21.81)	11,015 (21.96)	11,355 (22.63)	
No recorded	142,996 (71.26)	36,735 (73.23)	35,809 (71.38)	35,507 (70.78)	34,945 (69.66)	
Drinking status (%)						< 0.001
Current	1284 (0.64)	292 (0.58)	344 (0.69)	354 (0.71)	294 (0.59)	
Ever	8649 (4.31)	1764 (3.52)	2110 (4.21)	2363 (4.71)	2412 (4.81)	
Never	47,729 (23.79)	11,374 (22.67)	11,901 (23.72)	11,939 (23.8)	12,515 (24.95)	
No recorded	142,996 (71.26)	36,735 (73.23)	35,809 (71.38)	35,507 (70.78)	34,945 (69.66)	
Family history of diabetes (%)						< 0.001
No	196,452 (97.90)	48,785 (97.25)	49,059 (97.8)	49,193 (98.07)	49,415 (98.5)	
Yes	4206 (2.10)	1380 (2.75)	1105 (2.2)	970 (1.93)	751 (1.5)	

*BMI* body mass index, *HDL-C* high-density lipoprotein cholesterol, *LDL* low density lipoprotein, *FPG* fasting plasma glucose, *TC* total cholesterol, *TG* triglyceride, *SBP* systolic blood pressure, *DBP* diastolic blood pressure, *Cre/BW* creatinine to body weight, *Cre* concentration of creatinine, *AST* aspartate aminotransferase, *ALT* alanine aminotransferase, *BUN* blood urea nitrogen, *T2DM* type 2 diabetes mellitus, *CI* confidence interval, *HR* hazard ratio

### Univariate analysis for T2DM

Table 2 presents the results of the univariate analysis of the association between risk factors and incident T2DM. Using the univariate Cox proportional hazard model, we identified age, height, weight, SBP, DBP, FPG, TC, TG, LDL, ALT, AST, BUN, Cre, and family history of diabetes as being positively related to future risk of diabetes. Moreover, never smoking and HDL-C levels were negatively correlated. Furthermore, compared to men, women showed a lower risk of diabetes. Ever smoking was not associated with T2DM compared to current smoking.

**Table 2:** Univariate analysis for type 2 diabetes mellitus

	HR (95%CI)	P-value
Age (years)	1.07 (1.06,1.07)	< 0.001
Height (cm)	1.00 (1.00,1.01)	0.029
Weight (kg)	1.05 (1.05,1.05)	< 0.001
SBP (mmHg)	1.04 (1.04,1.04)	< 0.001
DBP (mmHg)	1.05 (1.04,1.05)	< 0.001
FPG (mmol/L)	10.43 (9.98,10.90)	< 0.001
TC (mmol/L)	1.43 (1.39,1.47)	< 0.001
TG (mmol/L)	1.26 (1.25,1.28)	< 0.001
HDL-C (mmol/L)	0.53 (0.47,0.61)	< 0.001
LDL (mmol/L)	1.41 (1.34,1.49)	< 0.001
ALT (U/L)	1.00 (1.00,1.00)	< 0.001
AST (U/L)	1.01 (1.01,1.01)	< 0.001
BUN (mmol/L)	1.24 (1.21,1.26)	< 0.001
Cre (mg/dl)	1.67 (1.56,1.78)	< 0.001
Sex (%)		
Men	Ref	
Women	0.48 (0.45,0.52)	< 0.001
Smoking status (%)		
Current	Ref	
Ever	0.81 (0.64,1.04)	0.097
Never	0.44 (0.38,0.49)	< 0.001
Not recorded	0.59 (0.53,0.65)	< 0.001
Drinking status (%)		
Current	Ref	
Ever	0.46 (0.38,0.54)	< 0.001
Never	0.42 (0.36,0.49)	< 0.001
Not recorded	0.49 (0.37,0.65)	< 0.001
Family history of diabetes (%)		
No	Ref	
Yes	1.7 (1.45,1.98)	< 0.001

*Cre/BW* creatinine to body weight, *BMI* body mass index, *HDL-C* high-density lipoprotein cholesterol, *LDL* low density lipoprotein, *FPG* fasting plasma glucose, *TC* total cholesterol, *TG* triglyceride, *SBP* systolic blood pressure, *DBP* diastolic blood pressure, *TyG-BMI* triglyceride glucose-body mass index, *Cre* concentration of creatinine, *AST* aspartate aminotransferase, *ALT* alanine aminotransferase, *BUN* blood urea nitrogen, *T2DM* type 2 diabetes mellitus, *CI* confidence interval, *HR* hazard ratio

**Fig. 2:**
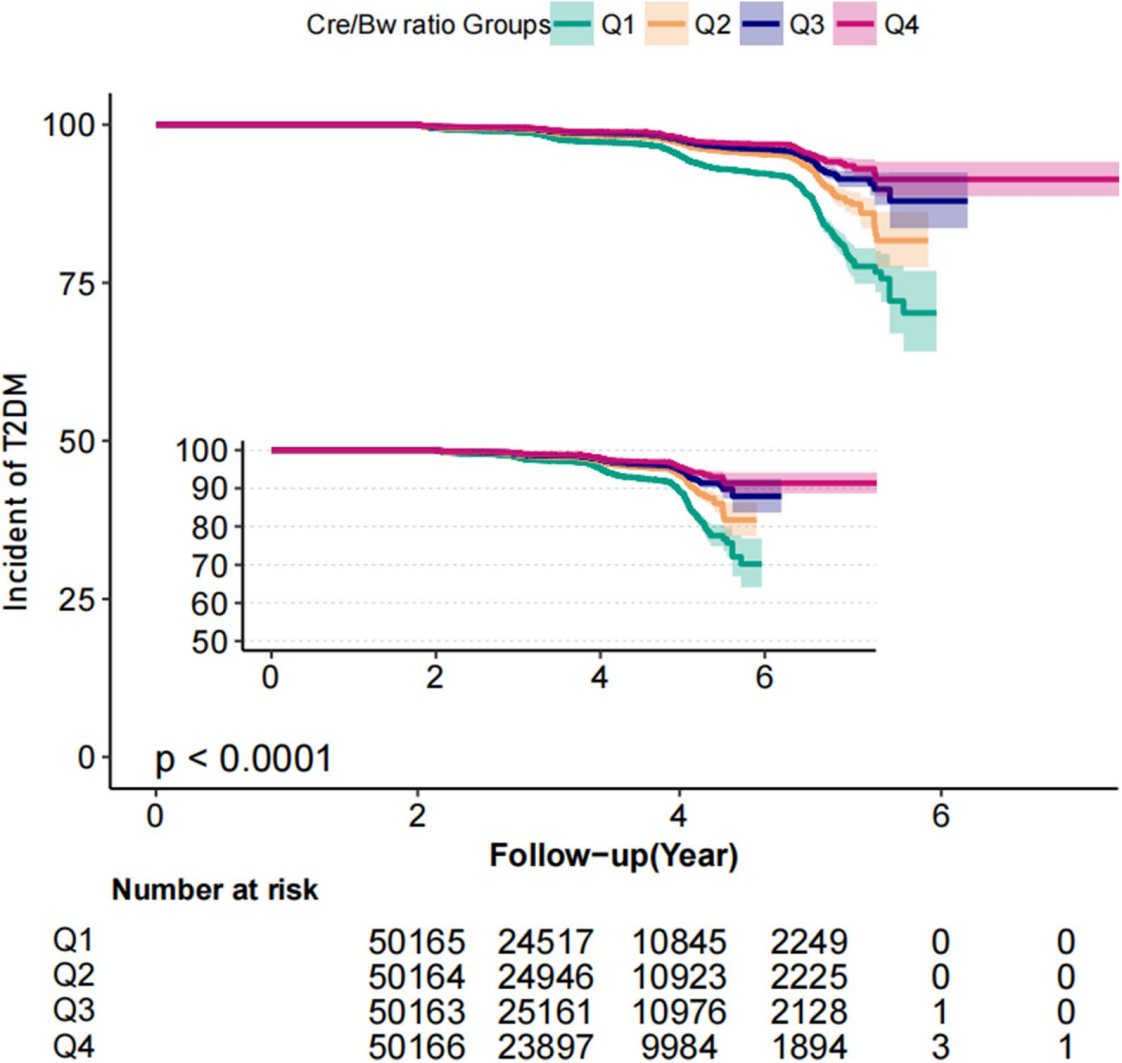
Kaplan–Meier analysis of T2DM risk according to Cre/Bw ratio

As shown in Fig. 2, the Kaplan–Meier curve revealed that the cumulative risk of incident diabetes was markedly different among the Cre/BW ratio quartiles (log-rank test, P < 0.001) and decreased gradually with an increase in Cre/BW ratio, resulting in a maximum risk of diabetes in the lowest quartile.

### Relationship between Cre/BW ratio and incident T2DM in different models.

We used Cox proportional hazard models to assess the independent effects of Cre/BW ratio (× 100) on the risk of incident T2DM (univariate and multivariate Cox proportional hazard models). Table 3 presents the effect sizes [hazard ratio (HR) and 95% confidence intervals (95% CI)]. According to the non-adjusted model, there was a negative relationship between the Cre/BW ratio (× 100) and incident T2DM, with an HR of 0.21 (0.18, 0.24). After adjusting for age, sex, height, FPG, SBP, DBP, TC, TG, LDL, HDL-c, BUN, AST, ALT, drinking status, smoking status, and family history of diabetes, the negative relationship between the Cre/BW ratio and incident T2DM did not change in the multivariate analysis [0.36 (0.31, 0.42]). Participants who had a Cre/BW ratio in the highest quartile versus the lowest quartile had a half-decreased risk in the odds of the development of T2DM [0.52 (0.47, 0.58]) (P for trend < 0.001).

**Table 3:** Relationship between Cre/BW ratio and incident T2DM in different models

Variable	Model 1		Model 2		Model 3	
	HR (95% CI)	P value	HR (95% CI)	P value	HR (95% CI)	P value
Cre/BW ratio (× 100)	0.21 (0.18 ~ 0.24)	< 0.001	0.11 (0.1 ~ 0.13)	< 0.001	0.36 (0.31 ~ 0.42)	< 0.001
Cre/BW ratio quartiles						
Q1	Ref		Ref		Ref	
Q2	0.6 (0.56 ~ 0.65)	< 0.001	0.55 (0.51 ~ 0.59)	< 0.001	0.72 (0.66 ~ 0.78)	< 0.001
Q3	0.48 (0.44 ~ 0.52)	< 0.001	0.39 (0.36 ~ 0.43)	< 0.001	0.64 (0.58 ~ 0.7)	< 0.001
Q4	0.4 (0.36 ~ 0.44)	< 0.001	0.27 (0.24 ~ 0.29)	< 0.001	0.52 (0.47 ~ 0.58)	< 0.001
P for trend		< 0.001		< 0.001		< 0.001

Model 1 was not adjusted. Model 2 was adjusted for age and sex. Model 3 was adjusted for age, sex, height, FPG, SBP, DBP, TC, TG, LDL, HDL-C, BUN, AST, ALT, drinking status, smoking status and family history of diabetes. *Cre/BW* creatinine to body weight

### Dose–response association between Cre/BW ratio and incident T2DM

**Fig. 3:**
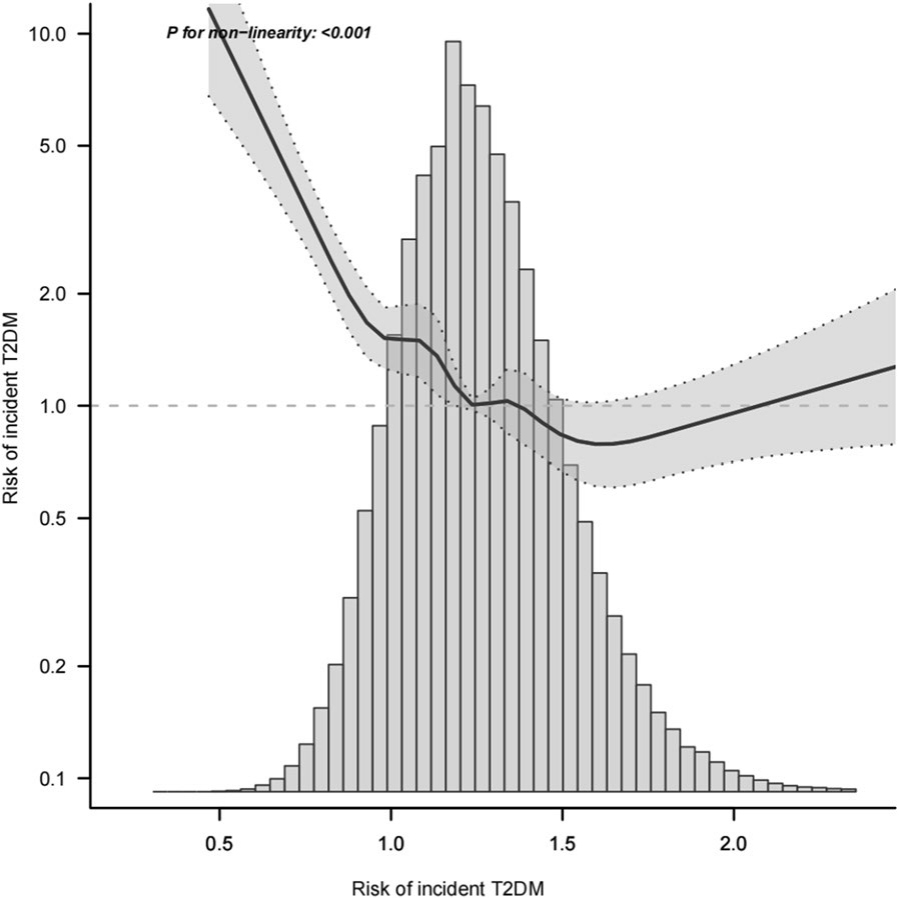
A nonlinear relationship of Cre/BW ratio (× 100) with risk of incident T2DM. Note: the model was adjusted for age, sex, height, FPG, SBP, DBP, TC, TG, LDL, HDL-c, BUN, AST, ALT, drinking status, smoking status and family history of diabetes

We used the restricted cubic spline curves to evaluate the dose–response relationship between Cre/BW ratio (× 100) and incident T2DM. After adjusting for potential confounders, an L-shaped nonlinear relationship between the Cre/BW ratio (× 100) and T2DM was observed (Fig. 3). The risk of incident T2DM was negatively correlated with the Cre/BW ratio (× 100). The risk of developing T2DM decreased significantly with Cre/BW ratio (× 100) until it peaked at 0.86 [0.01 (0.00,0.10), P < 0.001]. When the Cre/BW ratio (× 100) was between 0.86 and 1.36, the risk ratio for developing T2DM was 0.22 (0.12,0.38), P < 0.001. However, when the Cre/BW ratio (× 100) was > 1.36, the curve indicating the risk of developing T2DM became significantly flatter as the Cre/BW ratio (× 100) increased, with an HR of 0.73 (0.29,1.8), P = 0.49 (Table 4).

**Table 4:** Threshold effect analysis of Cre/BW ratio on incident T2DM

Outcome	HR (95% CI)	P value
One-line linear regression model	0.36 (0.31 ~ 0.42)	< 0.001
Three-piecewise linear regression model		
Cre/BW ratio < 0.86	0.01 (0.00,0.10)	< 0.001
Cre/BW ratio 0.86–1.36	0.22 (0.12,0.38)	< 0.001
Cre/BW ratio ≥ 1.36	0.73 (0.29,1.8)	0.49
Log-likelihood ratio test		< 0.001

Adjusted for age, sex, height, FPG, SBP, DBP, TC, TG, LDL, HDL-C, BUN, AST, ALT, drinking status, smoking status and family history of diabetes

In Fig. 3, the solid line curve indicates the estimated risk of incident T2DM. The dotted lines represent point-wise 95% CIs adjusted for age, sex, height, FPG, SBP, DBP, TC, TG, LDL, HDL-C, BUN, AST, ALT, drinking status, smoking status, and family history of diabetes. As the Cre/BW ratio (× 100) increased, the slope of the curve showed a decreasing L-shaped trend. In addition, we found that the Cre/BW ratio remained non-linearly and negatively correlated with T2DM after removed the upper and the lower 0.25% of the data to fit the curves. (Additional file 1: Fig. S1).

### Subgroup analyses

The participants were divided into subgroups according to age, sex, BMI, BUN, smoking status, drinking status, and family history of diabetes. Stratified analyses were performed to further explore the potential modification effect of the association between Cre/BW ratio (× 100) (< 0.86, 0.86–1.36 and ≥ 1.36) and the risk of incident T2DM by the various subgroups. (Table 5). The results showed that the association between Cre/BW ratio (× 100) and incident T2DM was stable in the different subgroups. A stronger inverse association between Cre/BW ratio (× 100) and the risk of incident T2DM was found in men (men vs. women, P interaction = 0.026); younger people [< 40 (tertile 1) vs. 40– < 60 vs. ≥ 60 years old, P interaction < 0.001]; those with lower BUN (≤ 7.1 vs. > 7.1 mmol/L, P interaction = 0.009); people who smoked more frequently [current (quartile 1) vs. ever vs. never vs. not-recorded, P interaction = 0.048]; and people who drank more frequently [current (quartile 1) vs. ever vs. never vs. not-recorded, P interaction = 0.049] (Table 5). Family history of diabetes (no vs. yes) did not significantly modify the association between Cre/BW ratio (× 100) and the risk of incident T2DM (Table 5) (Additional file 2: Fig. S2, Additional file 3: Table S1).

**Table 5:** Subgroup analyses of the association between Cre/BW ratio and incident T2DM

Subgroup	N1 (< 0.86)	N2 (≥ 0.86, < 1.36)	N3 (≥ 1.36)	Cre/BW ratio (× 100)	P. for. interaction
Sex					0.026
Women					
	1 (Ref)	0.61 (0.52 ~ 0.71)	0.48 (0.38 ~ 0.6)	0.44 (0.34 ~ 0.58)	
		< 0.001	< 0.001	< 0.001	
Men					
	1 (Ref)	0.57 (0.5 ~ 0.65)	0.4 (0.34 ~ 0.46)	0.34 (0.29 ~ 0.4)	
		< 0.001	< 0.001	< 0.001	
Age					< 0.001
< 40					
	1 (Ref)	0.48 (0.38 ~ 0.6)	0.22 (0.16 ~ 0.31)	0.09 (0.06 ~ 0.13)	
		< 0.001	< 0.001	< 0.001	
≥ 40, < 60					
	1 (Ref)	0.73 (0.6 ~ 0.89)	0.43 (0.36 ~ 0.52)	0.33 (0.27 ~ 0.41)	
		0.002	< 0.001	< 0.001	
≥ 60					
	1 (Ref)	0.84 (0.68 ~ 1.03)	0.63 (0.5 ~ 0.78)	0.7 (0.57 ~ 0.87)	
		0.089	0.655	0.001	
BUN					0.009
≤ 7.1					
	1 (Ref)	0.6 (0.54 ~ 0.66)	0.43 (0.38 ~ 0.49)	0.34 (0.3 ~ 0.4)	
		< 0.001	< 0.001	< 0.001	
> 7.1					
	1 (Ref)	0.33 (0.19 ~ 0.56)	0.21 (0.12 ~ 0.38)	0.54 (0.35 ~ 0.83)	
		0.309	0.035	0.005	
Smoking status					0.048
Current					
	1 (Ref)	0.37 (0.26 ~ 0.53)	0.2 (0.13 ~ 0.32)	0.18 (0.1 ~ 0.3)	
		< 0.001	< 0.001	< 0.001	
Ever					
	1(Ref)	0.52 (0.2 ~ 1.35)	0.27 (0.08 ~ 0.87)	0.11 (0.03 ~ 0.42)	
		0.181	0.029	0.002	
Never					
	1(Ref)	0.5 (0.39 ~ 0.64)	0.38 (0.28 ~ 0.52)	0.37 (0.26 ~ 0.53)	
		< 0.001	< 0.001	< 0.001	
No recorded					
	1 (Ref)	0.81 (0.71 ~ 0.91)	0.69 (0.61 ~ 0.79)	0.4 (0.34 ~ 0.47)	
		< 0.001	< 0.001	< 0.001	
Drinking status					0.049
Current					
	1 (Ref)	0.30 (0.10 ~ 0.92)	0.27 (0.07 ~ 1.1)	0.11 (0.02 ~ 0.67)	
		0.035	0.068	0.017	
Ever					
	1 (Ref)	0.34 (0.21 ~ 0.55)	0.20 (0.10 ~ 0.38)	0.21 (0.09 ~ 0.46)	
		< 0.001	< 0.001	< 0.001	
Never					
	1 (Ref)	0.50 (0.40 ~ 0.62)	0.34 (0.26 ~ 0.45)	0.3 (0.22 ~ 0.41)	
		< 0.001	< 0.001	< 0.001	
No recorded					
	1(Ref)	0.63 (0.57 ~ 0.71)	0.46 (0.4 ~ 0.53)	0.4 (0.34 ~ 0.47)	
		< 0.001	< 0.001	< 0.001	
Family history of diabetes					0.997
No					
	1(Ref)	0.59 (0.53 ~ 0.66)	0.42 (0.37 ~ 0.48)	0.36 (0.31 ~ 0.42)	
		< 0.001	< 0.001	< 0.001	
Yes					
	1(Ref)	0.54 (0.34 ~ 0.85)	0.37 (0.19 ~ 0.7)	0.27 (0.12 ~ 0.59)	
		0.008	0.002	0.001	

*Cre/BW* creatinine to body weight, *BUN* blood urea nitrogen, *T2DM* type 2 diabetes mellitus, *CI* confidence interval, *HR* hazard ratio

## Discussion

In this population-based retrospective cohort study, Cre/BW ratio was found to be negatively associated with the incidence of T2DM, independent of age, sex, height, FPG, SBP, DBP, TC, TG, LDL, HDL-C, BUN, AST, ALT, drinking status, smoking status, and family history of diabetes. We further revealed an L-shaped nonlinear relationship between the Cre/BW ratio and the risk of T2DM. The relationship was characterized as follows: the risk of developing T2DM decreased significantly with Cre/BW ratio (× 100) until it peaked at 0.86 [0.01 (0.00,0.10), P < 0.001]. When the Cre/BW ratio (× 100) was between 0.86 and 1.36, the risk ratio for developing T2DM was 0.22 (0.12,0.38), P < 0.001. However, when the Cre/BW ratio (× 100) was > 1.36, the curve showing the risk of developing T2DM became significantly flatter.

The skeletal muscle, which accounts for approximately 40% of the body weight, is the most significant metabolic organ and plays a vital role in the homeostasis regulation of glycometabolism and lipid metabolism throughout the body [18, 19]. Skeletal muscles can store about 60–80% of postprandial glucose [18]. During hyperinsulinemia-euglycemia episodes, the skeletal muscles account for 80–90% of the blood glucose [20]. Lower muscle mass may reduce the blood glucose intake [21]. In addition, muscle mass is negatively associated with IR and prediabetes, and IR is a critical pathogenic mechanism of diabetes [22]. Therefore, muscle quality is closely related to the occurrence of diabetes. It is well known that height-adjusted SMI, defined as appendicular skeletal muscle mass/height squared, is an important marker for sarcopenia [23]. However, an increase in both muscle mass and fat mass could lead to weight gain [24]. Thus, the proportion of muscle mass per body weight is essential. Multiple studies have reported that weight-adjusted appendicular skeletal muscle mass, but not height-adjusted SMI, is associated with cardiometabolic risk factors and IR [22, 25–28]. Moreover, weight-adjusted appendicular skeletal muscle mass is associated with incident diabetes [12], metabolic syndrome [29], and non-alcoholic fatty liver disease (NAFLD) [30–32]. This is because low-weight-adjusted SMI is associated with an increase in visceral fat, which in turn is also associated with the occurrence of diabetes [33]. Cre is an alternative marker of skeletal muscle mass, and its levels are positively correlated with skeletal muscle mass [13, 34, 35]. Therefore, weight-adjusted SMI was positively associated with the Cre/BW ratio. In addition, in previous studies, Cre/BW ratios have been confirmed to be associated with an increased risk of NAFLD, which has been confirmed to be positively associated with diabetes [7, 36–38]. Based on these findings, the Cre/BW ratio is associated with the incidence of diabetes.

Recently, a study indicated that Cre/BW is associated with incident diabetes and proposed that it predicts future diabetes and NAFLD [14, 16]. A cohort study of 9,659 men and 7,417 women with a follow-up mean (SD) of 5.6 (3.5) and 5.4 (3.4) years, respectively, showed that, compared to participants in the highest quartile of Cre/BW, participants in the lowest quartile had a higher risk of diabetes (relative risk 0.42 (0.32–0.54, P < 0.001] in men and 0.55 (0.34–0.89, P = 0.014] in women), after adjusting for age, FPG, alcohol consumption, exercise, and smoking [14, 16]. Moreover, Hashimoto et al. found that Cre/BW was negatively associated with incident diabetes (adjusted HR 0.84, 95% CI 0.80–0.88, P < 0.001 for men and 0.88, 0.81–0.96, P = 0.003 for women) [14]. In addition, a cohort study of Chinese and Japanese found that Cre/BW was negatively correlated with the occurrence of NAFLD [16]. Based on the positive relationship between fatty liver and the onset of diabetes [36], these conclusions are consistent with our findings (Cre/BW as continuous variable: HR 0.44 [0.34, 0.58] for women; HR 0.34 [0.29, 0.4] for men; P for interaction = 0.026). Moreover, in our study, the relationship between Cre/BW and diabetes mellitus showed different HRs across different age groups. The HR and 95% CI for incident diabetes mellitus associated with Cre/BW were 0.09 (0.0.06,0.13) and 0.7 (0.57,0.87) for adults < 40 and > 60 years, respectively. In contrast, our study found a nonlinear relationship between serum Cre/BW and incident diabetes. As the Cre/BW ratio increased, the risk of developing T2DM decreased rapidly until the Cre/BW ratio (× 100) reached 0.86. When the Cre/BW ratio (× 100) was > 1.36, the curve indicating the risk of developing T2DM became significantly flatter. These results may be because fat is essential to the body, and the proportion of fat mass must be maintained in a stable state. However, at the same time, we also found that although the risk of diabetes decreased with the ratio increased, the risk of diabetes did not decrease significantly when the Cre/BW ratio (× 100) was ≥ 1.36. These results suggest Cre/BW ratio as a potentially viable predictor in people who want to prevent diabetes through weight loss. Further, the results suggest that prevention of T2DM development by aggressive muscle mass gain and weight loss is only optimal when the Cre/BW ratio (× 100) is < 0.86 and is slightly less effective when the Cre/BW ratio (× 100) is between 0.86 and 1.36. However, when the Cre/BW ratio (× 100) is > 1.36, increasing muscle mass and reducing body weight may not be as effective in preventing the onset of T2DM. When the Cre/BW ratio (× 100) was increased by 1 unit, the incidence of T2DM decreased by 99%. However, when the Cre/BW ratio (× 100) exceeded 1.36, for every 1 unit increase in the Cre/BW ratio (× 100) the T2DM incidence only decreased by 27%; further research is needed to verify this.

Our study had several strengths. (1) Compared to previous similar studies, our study had a relatively large sample size; (2) correlation between the Cre/BW ratio and T2DM was performed in China for the first time; and (3) To reduce the contingency of the results and improve the robustness, we treated the Cre/BW ratio as continuous and classification variables with univariate and multivariate Cox proportional hazard models, and found saturation between the Cre/BW ratio and incidence of T2DM.

In the subgroup analysis, we used stratified linear regression models and likelihood ratio tests to identify modifications and interactions, to obtain stable results in the different subgroups. However, in adults aged < 40 years, we found that the risk of developing T2DM decreased more significantly with an increased Cre/BW ratio. Correspondingly, people > 60 years old did not have as high diabetes prevention effects through weight loss and controlling of the body fat rates as did people < 40 years old.

There are some limitations to our study. (1) The participants were all Chinese. Therefore, the universality and extrapolation of this study results are weak. However, one-quarter of the global population with diabetes live in China. Our data were obtained from multiple centers in China with a wide geographical region and age range, making the results widely applicable to the Chinese population and even the global Chinese people. Nevertheless, further research on the Cre/BW ratio in different populations needs to be evaluated, given the racial differences. (2) T2DM was not diagnosed using a two-hour oral glucose tolerance test, which may have resulted in an underestimation of the number of cases of diabetes. (3) Raw data were limited; hence, we could not adjust for waist circumference, education level, and the intensity and frequency of exercise, which may affect the relationship between Cre/BW ratio and incident T2DM. (4) The raw data did not include information on the types and amounts of foods and beverages (including all types of water) consumed during the 24 h before the examination, which may have affected the blood levels of TG and glucose. (5) Data were collected between 2004 and 2015, with a difference of almost ten years in between, for some individuals in this study. However, according to the median follow-up years used for the stratification, the association between the Cre/BW ratio and incident T2DM was stable. (6) In this study, we did not distinguish between type 1 and T2DM. However, since T2DM accounts for approximately 95% of all diabetes cases, our findings may represent the population with T2DM. (7) As the population in our study was mainly those with physical examination data, this might have resulted in an under-representation of the population with chronic kidney disease (CKD) and a population selection bias. Therefore, the relationship between the Cre/BW ratio and incident T2DM in the CKD population may not be well represented.

## Conclusions

In conclusion, an increase in the Cre/BW ratio was independently associated with a lower incidence of T2DM in this retrospective study during a 3.1-year follow-up of Chinese participants. The Cre/BW ratio is a negative and independent risk factor for T2DM events in the Chinese population. An L-shaped negative curvilinear association between Cre/BW ratio and incident T2DM was present, with a saturation effect predicted at 0.86 and 1.36 of Cre/BW ratio (× 100). Further studies are required to evaluate the predictive value of the Cre/BW ratio for T2DM.

## Supplementary Information

**Additional file 1: Figure S1.** A nonlinear relationship of Cre/BW ratio (× 100) with risk of incident T2DM removed the upper 0.25% of the data and the lower 0.25% of the data. Note: the model was adjusted for age, sex, height, FPG, SBP, DBP, TC, TG, LDL, HDL-c, BUN, AST, ALT, drinking status, smoking status and family history of diabetes.

**Additional file 2: Figure S2.** Nonlinear relationship of Cre/BW ratio (× 100) with risk of incident T2DM in each subgroup. A: Relationship of Cre/BW ratio (× 100) with risk of incident T2DM in Sex subgroup. B: Relationship of Cre/BW ratio (× 100) with risk of incident T2DM in Age subgroup. C: Relationship of Cre/BW ratio (× 100) with risk of incident T2DM in BUN subgroup. D: Relationship of Cre/BW ratio (× 100) with risk of incident T2DM in Smoking Status subgroup. E: Relationship of Cre/BW ratio (× 100) with risk of incident T2DM in Drinking Status subgroup. F: Relationship of Cre/BW ratio (× 100) with risk of incident T2DM in Family History subgroup. Note: the model was adjusted for age, sex, height, FPG, SBP, DBP, TC, TG, LDL, HDL-c, BUN, AST, ALT, drinking status, smoking status and family history of diabetes.

**Additional file 3: Table S1.** Relationship between creatinine and incident T2DM in different models.

## Data Availability

Data can be downloaded from the DRYAD database (http://www.Datadryad.org).

## Abbreviations

T2DM: Type 2 diabetes mellitus
IR: Insulin resistance
Cre/BW: Creatinine to body weight
SMI: Skeletal muscle mass index
FPG: Fasting plasma glucose
BMI: Body mass index
HDL-c: High-density lipoprotein cholesterol
LDL: Low density lipoprotein
TC: Total cholesterol
TG: Triglycerides
Cre: Serum creatinine
BUN: Blood urea nitrogen
AST: Aspartate aminotransferase
ALT: Alanine aminotransferase
SBP: Systolic blood pressure
DBP: Diastolic blood pressure
