# Supplementary figures and images for: Creatinine-to-body weight ratio is a predictor of incident diabetes: a population-based retrospective cohort study
Source: Diabetol Metab Syndr. 2022 Jan 15;14:7. doi: 10.1186/s13098-021-00776-8 (PMC8760680; doi:10.1186/s13098-021-00776-8)

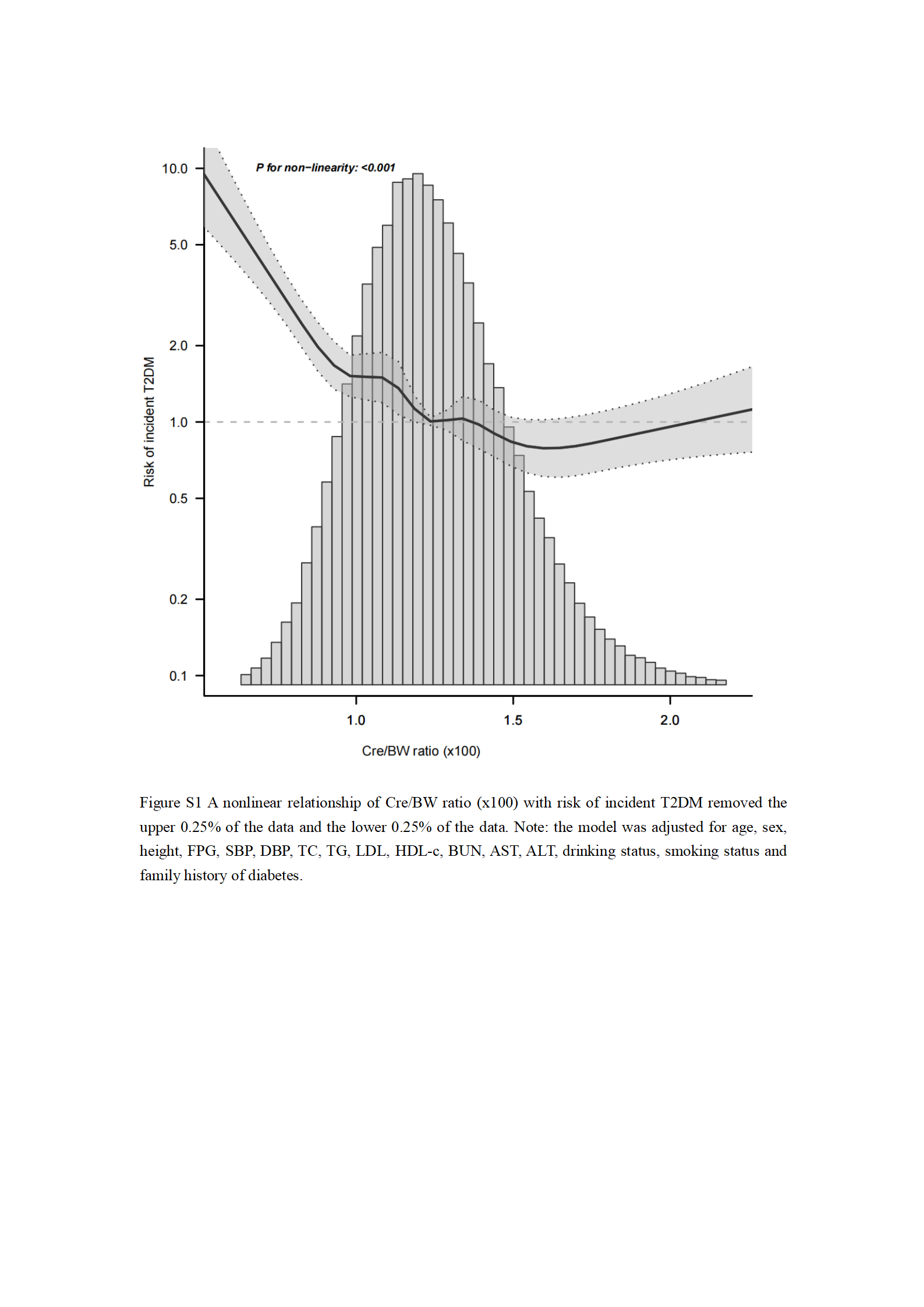

Supplement: Supplementary file 1 — Additional file 1: Figure S1. A nonlinear relationship of Cre/BW ratio (× 100) with risk of incident T2DM removed the upper 0.25% of the data and the lower 0.25% of the data. Note: the model was adjusted for age, sex, height, FPG, SBP, DBP, TC, TG, LDL, HDL-c, BUN, AST, ALT, drinking status, smoking status and family history of diabetes. [file 13098_2021_776_MOESM1_ESM.png]

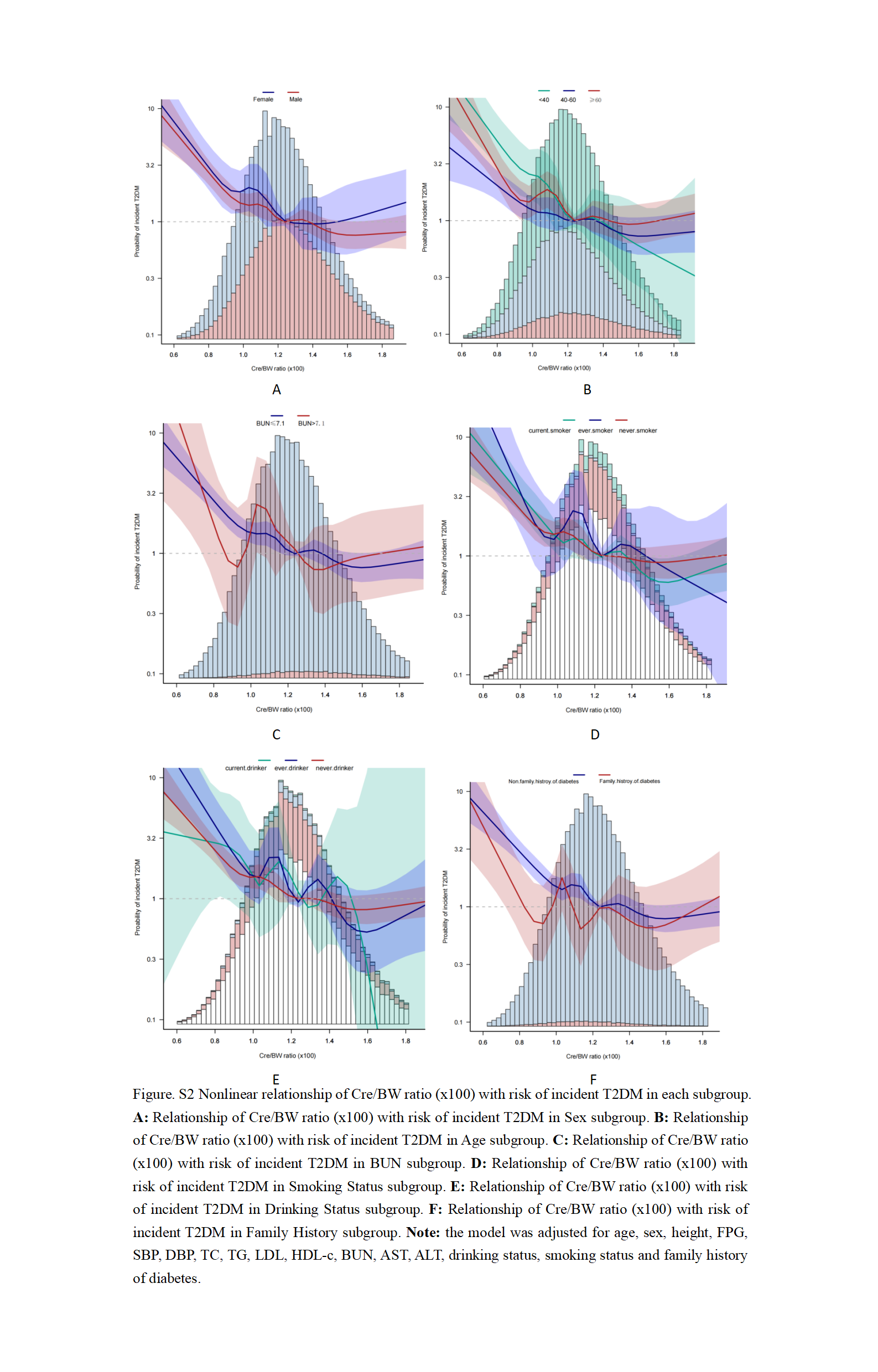

Supplement: Supplementary file 2 — Additional file 2: Figure S2. Nonlinear relationship of Cre/BW ratio (× 100) with risk of incident T2DM in each subgroup. A: Relationship of Cre/BW ratio (× 100) with risk of incident T2DM in Sex subgroup. B: Relationship of Cre/BW ratio (× 100) with risk of incident T2DM in Age subgroup. C: Relationship of Cre/BW ratio (× 100) with risk of incident T2DM in BUN subgroup. D: Relationship of Cre/BW ratio (× 100) with risk of incident T2DM in Smoking Status subgroup. E: Relationship of Cre/BW ratio (× 100) with risk of incident T2DM in Drinking Status subgroup. F: Relationship of Cre/BW ratio (× 100) with risk of incident T2DM in Family History subgroup. Note: the model was adjusted for age, sex, height, FPG, SBP, DBP, TC, TG, LDL, HDL-c, BUN, AST, ALT, drinking status, smoking status and family history of diabetes. [file 13098_2021_776_MOESM2_ESM.png]
