# Supplementary material for: Creatinine-to-body weight ratio is a predictor of incident diabetes: a population-based retrospective cohort study
Source: Diabetol Metab Syndr. 2022 Jan 15;14:7. doi: 10.1186/s13098-021-00776-8 (PMC8760680; doi:10.1186/s13098-021-00776-8)
Supplement: Supplementary file 3 — Additional file 3: Table S1. Relationship between creatinine and incident T2DM in different models. [file 13098_2021_776_MOESM3_ESM.docx]

| Table S1. Relationship between creatinine and incident T2DM in different models. | | | | | | |
| --- | --- | --- | --- | --- | --- | --- |
| Variable | model 1 | | model 2 | | model 3 | |
|  | HR (95% CI) | P value | HR (95% CI) | P value | HR (95% CI) | P value |
| creatinine | 1.67 (1.56~1.78) | <0.001 | 0.30 (0.24~0.39) | <0.001 | 0.50 (0.31~0.80) | 0.004 |
| Model 1 was not adjusted. Model 2 was adjusted for age and sex. Model 3 was adjusted for age, sex, height, FPG, SBP, DBP, TC, TG, LDL, HDL-C, BUN, AST, ALT, drinking status, smoking status and family history of diabetes.T2DM: type 2 diabetes mellitus; CI: confidence interval; HR: hazard ratio | | | | | | |
